# Centering Impact and Equity in the Next 50 Years of Eating Disorder Research

**DOI:** 10.1002/eat.24493

**Published:** 2025-06-25

**Authors:** Megan E. Mikhail, C. Blair Burnette

**Affiliations:** ^1^ Department of Psychiatry and Behavioral Sciences University of California San Francisco California USA; ^2^ Department of Psychology Michigan State University East Lansing Michigan USA

**Keywords:** disordered eating, eating disorders, equity, lived experience, research priorities

## Abstract

Over the past 50 years, there has been a significant increase in research on eating disorders, which is encouraging and necessary given the substantial mortality, morbidity, and personal and societal costs of these common conditions. However, improvements in prevention, treatment access, and outcomes have been slow, and research priorities have not always been aligned with the needs and interests of affected communities. In this commentary, we call on the field to think critically about how to maximize not only the volume but also the real‐world impact of the next 50 years of eating disorder research. In particular, we highlight the importance of creating a research ecosystem that nurtures methodological diversity and centers collaborative, participatory research in partnership with people with lived experience. We also underscore the importance of conducting inclusive research that makes space for the full diversity of eating disorder presentations and the people who experience them. Ultimately, we argue that creativity and commitment will be required along with shifts in training, academic incentives, and broader structures to realize the full potential of research to enhance the lives of those with eating disorders.

Eating disorders (EDs) are highly prevalent conditions with marked morbidity, mortality, and social and economic costs. In the United States alone, someone dies from an ED every 52 min (Streatfeild et al. 2021). Despite the many and potentially severe consequences of EDs, the overwhelming majority of affected people will never receive treatment. Among the minority who *do* receive treatment, many will not respond, many more will display a suboptimal response, and many will relapse or develop a chronic course. Accordingly, research into the etiology, maintenance, prevention, and treatment of these conditions is urgently needed.

Lee and Chi (2025) reviewed 50 years of ED research—from 1974 to 2024—to identify publication trends, themes, and public and academic engagement. Overall, the authors found that ED research output increased over time, especially after 2000, which is both encouraging and vital given the seriousness of these illnesses. However, improvements in real‐world conditions have failed to keep pace. The prevalence of EDs continues to rise, particularly post‐COVID‐19, which drove substantial increases in both the number of identified cases and severity of EDs among young people (as indexed by climbing rates of diagnoses and hospitalizations; Pastore et al. 2023). Meanwhile, advances in treatment access and outcomes have been modest, particularly for individuals with more severe presentations and longer duration of illness. For us as researchers and clinicians deeply invested in advancing the welfare of people with EDs, this raises a critical concern: what might we be missing that could meaningfully reduce the prevalence and progression of these disorders?

As we look ahead to the next 50 years of ED research, the field must reflect thoughtfully on how we can focus our efforts to maximize impact and substantively improve the lives of those affected. In other words, which topics and approaches will yield the most innovative, pragmatically actionable findings that resonate beyond the ivory tower? Answering this question will require listening closely to the priorities of impacted communities and the public more broadly. However, Lee and Chi's (2025) finding that only about 9% of the variance in online engagement with ED work could be explained by citation scores suggests there is currently a disconnect between what is most central to the academic research community and what matters most to those outside of it. Altmetric Attention Scores (AAS) are an imperfect measure of public engagement, particularly given that online engagement through social media is a relatively recent phenomenon not applicable to research conducted before the 2010s. Nevertheless, the misalignment suggested by this statistic is echoed by studies that have asked individuals with EDs and their supporters directly what they think should be prioritized in research. Individuals who are personally impacted consistently emphasize the importance of work that can improve the wellbeing of people with EDs in the here and now, including advancing and individualizing treatment with input from voices of lived experience, finding ways to prevent relapse, addressing trauma and comorbidity, and facilitating access to care (e.g., Obeid et al. 2020). While some of these areas are included in Lee and Chi's (2025) networks, they tend to be near the periphery (e.g., psychotherapy), and others are entirely absent (e.g., treatment access, trauma).

Remedying this discrepancy requires confronting the fact that traditional academic frameworks and incentive structures do not always facilitate, and at times implicitly impede, publicly engaged work that addresses the immediate needs of people with EDs. For example, Lee and Chi (2025) show that ED‐related publications are more likely to be cited if they address areas of basic biological science that are often further removed from on‐the‐ground treatment realities (e.g., neurosciences, endocrinology and metabolism). Given that citations are an essential currency in academic careers, this implies a tacit valuing of this type of research over other forms of scholarship that may be more immediately actionable. Basic biological research is undoubtedly valuable—it contributes meaningfully to our understanding of EDs and uncovers mechanisms that may eventually facilitate treatment advances. However, the preferential value placed on this type of work within academia does not necessarily align with its real‐world impact, which can be distant and uncertain.

To sustain a vibrant research ecosystem, we must foster an environment where a diversity of research approaches can not only flourish but are intentionally cultivated. Research that targets direct change, such as community based participatory research (CBPR), is an essential complement to basic science that can yield more immediate advances in response to current community needs. Yet “publish or perish” academic settings are often inhospitable to this kind of work, which requires a substantial investment of time and emotional energy to build relationships and navigate differences in knowledge, perspective, and social position. Community‐based approaches also require a fundamental shift in how we conceptualize the research process, from a model in which the scientist is the sole generator of knowledge and the participant is a passive subject to one that centers knowledge co‐creation. Making space for this kind of research will require deliberate effort on the part of organizations, funding agencies, and individual researchers, particularly those in leadership positions who have the leverage and influence to institute broader change. Nevertheless, building an environment that fosters community‐engaged research is not unprecedented or unachievable. Funders in several countries outside the United States, including the United Kingdom, have already begun to require substantive engagement with impacted communities in mental health research, demonstrating that systemic transformation is possible.

Simultaneously, it is worth thinking creatively about how even research in neurobiology or genetics might be designed to meaningfully incorporate community perspectives and emphasize translational relevance. We believe research in all domains can be strengthened by respectful, bidirectional conversation with voices of lived experience, and that siloing between research and practice is not inevitable for any area or methodological approach. For example, while research on genetically based temperamental traits associated with EDs could be pursued as a purely theoretical endeavor, its impact is amplified when it is conducted in the service of developing interventions that leverage neurobiological tendencies as strengths (Kaye et al. 2015). While some research areas may lend themselves to translation more easily than others (e.g., research on the role of emotions or cognitions in maintaining ED behaviors), the potential for any research to tangibly improve outcomes for people with EDs is enhanced when it is approached as a means of meeting community needs rather than purely as intellectual inquiry.

In addition to *what* and *how* we research, it is important to consider *who* is being included and centered in our scholarship, and who is marginalized or left out. It is notable that Lee and Chi (2025) identified no PubMed Medical Subject Headings (MeSH) terms related to aspects of identity or sociocultural position other than age and sex that reached their minimum threshold of 500 articles. While MeSH terms are imperfect and do not capture all aspects of identity or experience that might be relevant to ED research, terms such as “ethnic and racial minorities,” “socioeconomic status,” “social adversity”, and “persons with disabilities” could have been featured in ED‐related articles. Their absence suggests that even when people with minoritized identities have been included in ED research, the nuances of their specific experiences have rarely been foregrounded. Similarly, it is worth considering how the prevalence of some MeSH terms and absence of others reflects on the way subjects have often been framed within the field. For example, both “obesity” and “weight loss” reached the threshold for inclusion, but “weight prejudice” (or related terms, e.g., “weight‐based discrimination”) did not.

Conducting research that incorporates these populations and perspectives is important. It also requires intentional support across multiple levels in the field to be done sustainably because pursuing research in an understudied area can come with a professional cost. As an illustration of this latter point, while “male” reached the threshold for inclusion in Lee and Chi (2025), research focused on male populations incurred a citation penalty (indicated by the negative association between the MeSH term “male” and citations). Rather than a deliberate snub of male‐related research, this finding likely reflects the reality that fewer papers are published in areas that have been historically marginalized, leading to fewer citations of other work in the same domain. Addressing this issue requires structural change, including targeted efforts within journals (e.g., special issues focused on minoritized populations), recruitment of and social and instrumental support for researchers from marginalized backgrounds, greater emphasis within training contexts on the scientific importance of understanding human diversity, and revised academic evaluation metrics that better capture the value of equity‐focused research. It will also require commitment and courage, particularly for researchers within the United States, who are currently facing unprecedented political headwinds in pursuing representative, inclusive, and equitable research.

As Lee and Chi demonstrated, ED research has burgeoned over the last 50 years. While the growing research attention to these serious disorders is heartening, we also must reckon with the fact that 50 years of science have not yielded meaningful reductions in their prevalence, with more people than ever experiencing these life‐limiting illnesses. Though there have been some important advances in treatment, many people continue to suffer for years without remission due to issues of both access and efficacy. Further, these 50 years of research have kept some of the groups at highest risk for EDs (and subsequently negative outcomes) on the margins, including people from minoritized racial backgrounds, sexual and gender minorities, and individuals experiencing socioeconomic disadvantage. We argue that fundamental shifts in the priorities and conduct of ED research are needed to improve outcomes and shrink disparities. The research that garners the most scientific attention and accolades via citations and funding may not always represent the work most likely to shift the tide. Though systemic and institutional change will likely be arduous, incorporating community perspectives into research (from bench to bedside) and intentionally including and centering populations largely excluded from the last 50 years of ED research are actionable steps we can, and we argue *must*, take now. What is at stake is not only scientific progress, but the lives and well‐being of those our field exists to serve.

## Author Contributions

**Megan E. Mikhail:** conceptualization, writing – original draft, writing – review and editing. **C. Blair Burnette:** conceptualization, writing – original draft, writing – review and editing.

## Conflicts of Interest

The authors declare no conflicts of interest.

## Data Availability

Data sharing is not applicable to this article as no new data were created or analyzed in this study.
